# Watching Ion Channels on the Move

**DOI:** 10.1093/function/zqac072

**Published:** 2022-12-30

**Authors:** Luis A Pardo

**Affiliations:** Max Planck Institute for Multidisciplinary Sciences, Oncophysiology Group, Max Planck Institute for Multidisciplinary Sciences, City Campus, Hermann-Rein-Str. 3, 37075 Göttingen, Germany

Ion channels remain fascinating molecular machines implicated in virtually every cellular function. Their activity can be studied in deep detail using biophysical techniques down to the single-molecule level. However, as large hydrophobic proteins embedded in a lipidic environment, their structure has traditionally been very difficult to study. Cryo-EM approaches have boosted our knowledge in the last few years, expanding the collection of resolved structures almost on a weekly basis. Yet, there are still open questions regarding the structure-function of the channels that are now starting to find answers.

Ion channels react rapidly to a wide range of stimuli, opening a pathway for the flow of ions across the membrane. The coupling of the stimulus to the opening of the gate can be studied in ligand-gated channels by comparing the structures of the ligand-bound and unbound channels. Still, such a comparison is more difficult to achieve when the channel responds to physical rather than chemical stimuli, as is the case of voltage-gated channels. The molecular principles of voltage-dependent gating of ion channels have been known for four decades. The mechanism consists, in essence, of the movement of some parts of the protein (the voltage-sensing domains) relative to others. The displacement results in a conformational change that produces the opening of the gate, but the intimate molecular mechanisms linking both events remain only partly known in many cases. Although the problem might appear like an academic discussion for experts at first glance, it has many practical implications. On the one hand—mainly in nonexcitable cells—it is often the movement of the voltage sensor, and not the flow of ions through the channel that determines the cells biological functions. The interaction of Kv11.1 (HERG) with β-integrin is a prominent example of this situation^1^ that offers an opportunity to target tumor cells without altering cardiac function, which is the risk associated with HERG inhibition. On the other hand, the high degree of similarity between channels hinders the structure-based design of novel specific chemical modulators that can be used in therapy. Still, structurally close channels often have very different gating properties. If the parts of the molecule and the intramolecular arrangements determining such differences are elucidated, it should be possible to reach specificity by targeting those regions. The structures of ion channels were typically resolved in crystals, micelles, or lipid nanodiscs, where no membrane potential was present. Therefore, the voltage sensor can be assumed to be in the conformation corresponding to a 0 mV membrane potential, which is open in most cases. To be able to compare the open and closed conformations and then model the transition between the two structures by, for example, molecular dynamics simulations, the conformation of the channel under a negative membrane potential needed to be resolved. The closed conformation was mimicked by mutagenesis (generating “always closed” variants),^2^ by chemical modifications such as metal cross-linking of engineered cysteines,^3^ and other approaches. However, the genuine voltage-dependent resting state under hyperpolarized potential had not been achieved. Recent work^4^ has finally overcome such limitations by reconstituting Kv10.1 voltage-gated channels in lipid vesicles. A voltage gradient was created by establishing a gradient of potassium concentration and a selective permeability for the ion much in the way nature does in cells. The vesicles were produced in a solution rich in potassium and then immersed in a buffer with low potassium in the presence of valinomycin. The flow of potassium along its concentration gradient generates a potential across the membrane of the vesicle in the range of -145 mV. Under such conditions, the voltage sensor will be in the deactivated conformation. A comparison between this structure and those obtained at 0 mV gives an idea of the displacement of the voltage sensor domains.

Although there is still room for improvement because the structure resolved is not that of the permeating channel but of a complex with calmodulin, which acts as a channel inhibitor, this kind of experiment will not only advance our understanding of the intramolecular interactions during voltage-dependent gating. They also provide information on the rearrangements of the membrane lipids induced by the displacement of the voltage-sensing domain, which are important modulators of channel function, and on the changes of interactions with partner proteins. The functional channels are integrated into larger supramolecular complexes composed of different proteins, often containing more than one channel type. Because the function of the channels can be measured very accurately, they are a proxy for studying the operation of the whole complex. Refinement of the cryo-EM technology itself and the use of elegant reconstitution approaches has allowed resolving structures of such large supramolecular complexes of channels and accessory/modulatory proteins (eg^5–8^). The channelosome is at the end of the day, the structure in charge of the physiology.^9^ For example, patients suffering from Duchenne muscular dystrophia often present abnormal cardiac function that can have fatal consequences. In animal models of the disease, ion channel expression and function are abnormal, although the channels are not directly affected by the mutations responsible for the condition. Restoring the so-called dystrophin-associated protein complex by transfection of a scaffolding protein into iPSC cardiomyocytes from affected individuals is able to revert the pathological action potential and correct the arrhythmic phenotype. These results highlight the importance of the correct composition and spatiotemporal distribution of the complexes and not only of the ion channels.^10^

In summary, the ion channel field is living a rebirth driven by structural information progressively closer to the native conformation of the complexes where channels operate. Together with rapidly advancing molecular dynamics simulations, improvements in biophysical techniques, and sophisticated models, our understanding of integrative ion channel physiology will certainly flourish in the coming years.

## References

[1] Forzisi E , Sesti F. Non-conducting functions of ion channels: the case of integrin-ion channel complexes. Channels. 2022;16(1):185–197. 35942524 10.1080/19336950.2022.2108565 PMC9364710

[2] Lenaeus MJ , Gamal El-Din TM, Ing C et al. Structures of closed and open states of a voltage-gated sodium channel. Proc Natl Acad Sci. 2017;114(15):E3051–E3060. 28348242 10.1073/pnas.1700761114 PMC5393245

[3] Ye W , Zhao H, Dai Y et al. Activation and closed-state inactivation mechanisms of the human voltage-gated K(V)4 channel complexes. Mol Cell. 2022;82(13):2427–2442.e4. 35597238 10.1016/j.molcel.2022.04.032 PMC9271590

[4] Mandala VS , MacKinnon R. Voltage-sensor movements in the Eag Kv channel under an applied electric field. Proc Natl Acad Sci. 2022;119(46):e2214151119. 36331999 10.1073/pnas.2214151119 PMC9674223

[5] Kschonsak M , Chua HC, Weidling C et al. Structural architecture of the human NALCN channelosome. Nature. 2022;603(7899):180–186. 34929720 10.1038/s41586-021-04313-5

[6] Zhou L , Liu H, Zhao Q, Wu J, Yan Z. Architecture of the human NALCN channelosome. Cell Discov. 2022;8(1):33. 35387979 10.1038/s41421-022-00392-4 PMC8986805

[7] Kang Y , Chen L. Structure and mechanism of NALCN-FAM155A-UNC79-UNC80 channel complex. Nat Commun. 2022;13(1):2639. 35550517 10.1038/s41467-022-30403-7 PMC9098444

[8] Kise Y , Kasuya G, Okamoto HH et al. Structural basis of gating modulation of Kv4 channel complexes. Nature. 2021;599(7883):158–164. 34552243 10.1038/s41586-021-03935-z PMC8566240

[9] Vierra NC , Trimmer JS. Ion Channel Partnerships: odd and not-so-odd couples controlling neuronal ion channel function. Int J Mol Sci. 2022;23(4):1953. 35216068 10.3390/ijms23041953 PMC8878034

[10] Jimenez-Vazquez EN , Arad M, Macias A et al. SNTA1 gene rescues ion channel function and is antiarrhythmic in cardiomyocytes derived from induced pluripotent stem cells from muscular dystrophy patients. Elife. 2022;11:e76576. 35762211 10.7554/eLife.76576 PMC9239678

